# Toxin Neutralization Using Alternative Binding Proteins

**DOI:** 10.3390/toxins11010053

**Published:** 2019-01-17

**Authors:** Timothy Patrick Jenkins, Thomas Fryer, Rasmus Ibsen Dehli, Jonas Arnold Jürgensen, Albert Fuglsang-Madsen, Sofie Føns, Andreas Hougaard Laustsen

**Affiliations:** 1 Department of Veterinary Medicine, University of Cambridge, Cambridge CB3 0ES, UK; tpj24@cam.ac.uk; 2 Department of Biochemistry, University of Cambridge, Cambridge CB3 0ES, UK; tf275@cam.ac.uk; 3 Department of Biotechnology and Biomedicine, Technical University of Denmark, DK-2800 Kongens Lyngby, Denmark; rasdeh@dtu.dk (R.I.D.); jjarnoldi@msn.com (J.A.J.); albertfuglsang@outlook.com (A.F.-M.); sofie.foens@gmail.com (S.F.); 4 Department of Biology, University of Copenhagen, DK-2200 København N, Denmark

**Keywords:** Snakebite envenoming, next-generation antivenom, toxin neutralization, alternative binding protein scaffolds, envenoming therapy, recombinant binding proteins, venom neutralization

## Abstract

Animal toxins present a major threat to human health worldwide, predominantly through snakebite envenomings, which are responsible for over 100,000 deaths each year. To date, the only available treatment against snakebite envenoming is plasma-derived antivenom. However, despite being key to limiting morbidity and mortality among snakebite victims, current antivenoms suffer from several drawbacks, such as immunogenicity and high cost of production. Consequently, avenues for improving envenoming therapy, such as the discovery of toxin-sequestering monoclonal antibodies against medically important target toxins through phage display selection, are being explored. However, alternative binding protein scaffolds that exhibit certain advantages compared to the well-known immunoglobulin G scaffold, including high stability under harsh conditions and low cost of production, may pose as possible low-cost alternatives to antibody-based therapeutics. There is now a plethora of alternative binding protein scaffolds, ranging from antibody derivatives (e.g., nanobodies), through rationally designed derivatives of other human proteins (e.g., DARPins), to derivatives of non-human proteins (e.g., affibodies), all exhibiting different biochemical and pharmacokinetic profiles. Undeniably, the high level of engineerability and potentially low cost of production, associated with many alternative protein scaffolds, present an exciting possibility for the future of snakebite therapeutics and merit thorough investigation. In this review, a comprehensive overview of the different types of binding protein scaffolds is provided together with a discussion on their relevance as potential modalities for use as next-generation antivenoms.

## 1. Introduction

Animal toxins have troubled humankind for millennia. These toxic proteins have evolved for use in prey subduction or as a natural defense mechanism to repel or kill predators by exerting hemotoxic, myotoxic, cytotoxic, and/or neurotoxic effects [1]. In turn, these toxicities result in various discomforting, debilitating, or lethal clinical manifestations in their victims (e.g., dizziness, ptosis, flaccid paralysis, coagulopathies, and hemorrhage) that often require timely treatment to prevent permanent damage [1]. For our ancestors an aggressive encounter with a venomous or poisonous animal would often have resulted in agonizing pain, permanent disability, or even death. However, intoxication and envenoming are not just a problem of the past. Venomous animals, including snakes, spiders, scorpions, caterpillars, sea anemones, jellyfish, lizards, fish, cone snails, bees, and wasps, even today kill over 150,000 people each year with the majority of deaths stemming from snakebites (81,000 to 138,000 deaths) [2]. The vast majority of snakebite-induced deaths occur in Asia (>57,600 deaths per year) and sub-Saharan Africa (>32,100 deaths per year) and has been unequivocally associated with poverty [3]. This has led to its recent reclassification as a Neglected Tropical Disease (NTD) by the World Health Organization [4]. Notably, the number of snakebite-induced mortalities in Africa and Asia is double that of all other NTDs combined (e.g., African trypanosomiasis, cholera, dengue hemorrhagic fever, leishmaniasis, Japanese encephalitis, and schistosomiasis) [2,5].

Currently, animal-derived antivenoms present the only effective treatment against envenoming from snakes and other species. The production of such antivenoms involves the immunization of horses or other animals (e.g., sheep and donkeys) with increasing amounts of the target venom(s). In response to this, the immune system of the production animals will give rise to polyclonal antibodies against the different venom proteins (of which many are toxins). Upon completion of the immunization process, the polyclonal antibodies can be isolated from the immunized animal plasma. Such plasma-derived antivenoms have been instrumental in saving lives and limbs of envenomed patients for over 120 years, since they were simultaneously developed by Césaire Auguste Phisalix, Gabriel Bertrand, and Albert Calmette in France in 1894 [6,7]. However, despite their long and successful clinical track record, the current plasma-derived antivenoms unfortunately present a number of drawbacks. The use of production animals drives up the cost of the antivenom, since they require a significant amount of space and costly veterinary care, amongst other things [8]. Consequently, this complicates the distribution of these antivenoms to the people most in need, since they typically do not have the economic means to cover the costs of their treatment. Furthermore, the heterologous nature of current antivenoms can lead to high immunogenicity and, consequently, a significant risk of adverse reactions (such as serum sickness and anaphylaxis) in treated snakebite victims [9,10,11,12,13].

A limited amount of innovation in envenoming therapy has occurred over the past century. However, we are now possibly seeing the dawn of the next chapter in the antibody-based therapies against animal envenomings. The development of human monoclonal antibodies (mAbs) by Georges Köhler & Cesar Milstein in 1975 dramatically expanded the scope and potential of antibody therapy [14]. mAbs now play a central role in the treatment of various forms of cancer, autoimmunity, and infectious disease, and are starting to find their use for toxin neutralization, i.e., in the treatment of *Staphylococcus aureus*’ dermonecrotizing alpha toxin [15], *Clostridium difficile*’s alpha toxin that induces nosocomial infectious diarrhea [16], the botulism-causing botulinum toxin type A [17], and also snake toxins [18].

**Figure 1:**
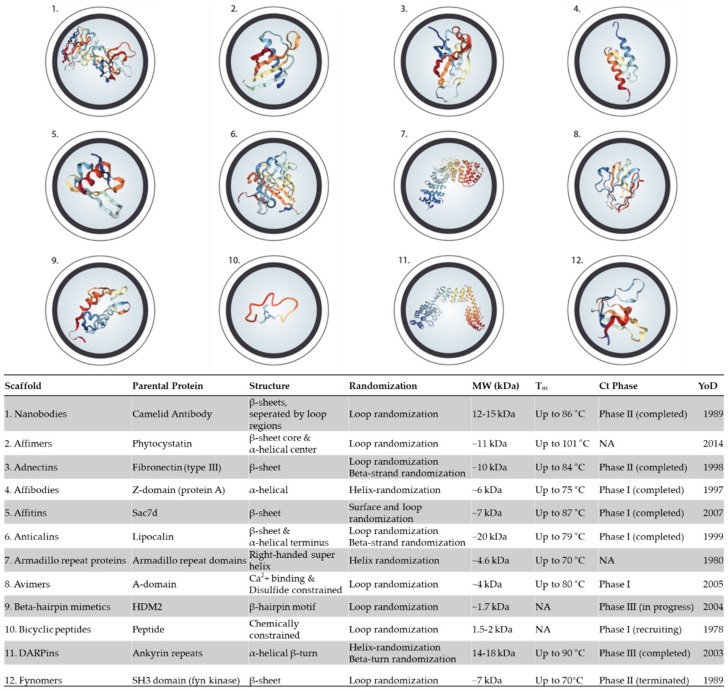
Overview of the structure, properties, and additional information of the alternative protein scaffolds covered in this review. The overview includes the parental proteins to each scaffold, the randomization strategies, the molecular weight (MW), maximum melting temperature (T_m_), most advanced clinical trial phase (Ct Phase; i.e., the most advanced clinical trial stage undergone by a particular scaffold), and the year of discovery (YoD). The figure also indicates where certain information was not available (NA). The figure was inspired by Vazquez-Lombardi et al., 2015 [25], and the scaffold images sourced from the Protein Databank (https://www.rcsb.org/).

Immunoglobulin G antibodies (IgGs) represent a well-validated and rapidly growing class of human therapeutics with long serum half-life, bivalency, and immune effector functions [19,20]. Particularly, recombinant IgG-based antivenoms have the potential to be safer and more efficacious snakebite therapies than current plasma-derived antivenoms. This arises from their compatibility with the human immune system and the possibility to only include antibodies that target medically relevant snake venom toxins in the antivenom mixture, rather than against all venom toxins, and other immunogenic components. However, there are some potential drawbacks connected to the use of IgGs for envenoming therapy, namely the large size of IgGs (~150 kDa) [21] and their complex structure [22], which might limit their rate of systemic distribution and require manufacturing processes based on mammalian cell cultivation [23,24]. As an alternative, small non-antibody scaffolds might be able to overcome some of the limitations of IgGs, while retaining many of their benefits. Such scaffolds include adnectins, affibodies, anticalins, and designed ankyrin repeat proteins (DARPins) among others, and are small, single-domain proteins that typically lack disulfide bonds, require no post-translational modifications, and can undergo straightforward multimerization (Figure 1) [25]. They present promising therapeutic scaffolds for antitoxin development, since their cost of production has the potential to be lower than the cost of production for IgGs (e.g., through low-cost microbial expression), and since their high stability could render cold chain unnecessary in their geographical distribution, which is a significant advantage particularly for envenomation treatment in rural areas where snakebite antivenom is needed the most [25]. Furthermore, the high levels of engineerability and likely improved tissue penetration for rapid distribution bear notable therapeutic potential.

In this review, we briefly introduce the different types of animal-induced poisonings and envenomings, discuss the current state-of-the-art in envenoming therapy in the clinic, succinctly present considerations toward the use of human antibodies, suggest and discuss alternative protein scaffolds to target toxins, as well as explore their history and properties. Finally, we briefly assess the potential impact of the implementation of alternative protein scaffolds as therapeutic agents against envenomings.

## 2. Poisonings and Envenomings

**Figure 2:**
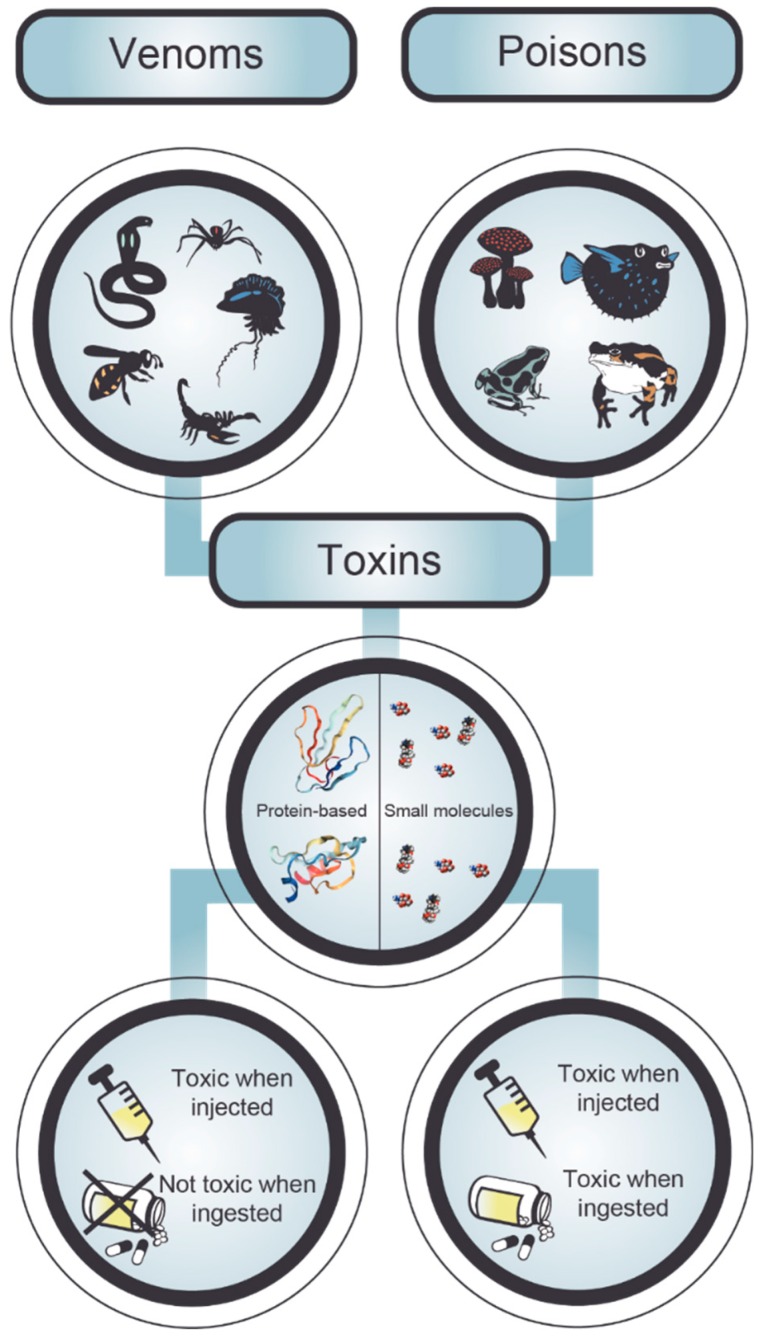
Venomous as well as poisonous animals produce and/or accumulate toxins. While venom toxins are protein based, poison toxins are mostly comprised of small organic molecules. Consequently, venoms are only toxic when injected, while poisons are toxic both when injected and ingested.

There are two major modalities by which humans are typically exposed to animal toxins, namely by poisoning or envenoming. Although the terms “poison” and “venom” are often used interchangeably, they do in fact have very different meanings and implications (Figure 2). The key distinguishing factor lies within the toxin delivery method of the animal [26]. Poison is absorbed or ingested by the affected individual, and therefore a poisonous animal can only deliver toxic compounds if another animal comes in contact with it or eats it (e.g., puffer fish, poison dart frog, cane toad) [26]. Venom, on the other hand, is always injected and consequently one can find a mechanism (e.g., stingers, fangs, etc.) to inject toxins directly into another creature in every venomous animal [26]. A further distinguishing factor, on a molecular level, is that venoms are typically protein-based, whereas poisons mostly contain small organic molecules [26]. Consequently, antibodies can be raised against venom toxins, whereas this is typically impossible for small molecules from poisons [18]. Venoms in particular are very complex, containing polypeptides, high-molecular-weight and low-molecular-weight proteins, amines, lipids, steroids, amino polysaccharides, quinones, glucosides, nucleosides, and free amino acids, as well as serotonin, histamine, and other substances [27]. The composition of a venom appears to reflect its function, resulting in defensive venoms, such as those from fish or bees, typically being relatively simple and primarily acting as immediate and extreme pain inducers [28,29,30]. Predatory venoms, on the other hand, are more complex (sometimes comprising over 100 different proteins) and often highly variable in composition and physiological effects, since they need to target various complex biological mechanisms within their prey [27]. Such diversity predisposes the venom composition of such species to vary even between individuals of the same species through random and selectively driven mutations. Consequently, such variation can also result in significant variation in overall venom toxicity and mode of action between closely related taxa [31], populations of a single species [32,33], sex-related differences in siblings [34], and ontogenetic variations in the lifetime of an individual [35]. This can have significant consequences for the efficacy of antivenoms for human therapy; antivenoms specifically developed towards a certain species might be ineffective, if the venoms used for immunization do not cover geographical and/or environmental variation in venom composition of that species. Notably, not all toxins in these venoms are of medical importance and need to be neutralized. Hence, for an effective treatment it is important to identify the clinically relevant toxins and to ensure that the therapeutic molecule(s) against these toxins can bind and neutralize their toxicity in the face of potential variation in venom compositions. However, to date, we have mostly relied on the ingenuity of the mammalian immune system in the context of serotherapy to mitigate the effects of envenomings; the ability to engineer targeted antibodies against specific toxins has only recently come within our reach, yet already holds significant promise.

## 3. Serotherapy against Intoxication

**Figure 3:**
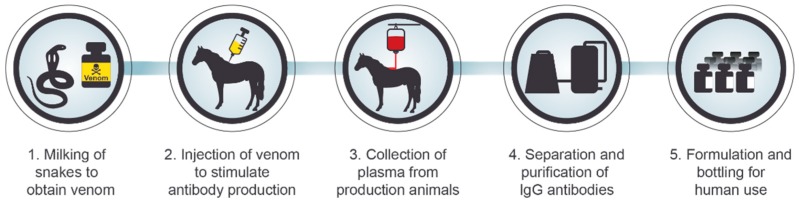
Schematic overview of the production process for current snake antivenoms. First, the venom needs to be manually extracted from the target species of snake(s); a process commonly known as “milking”. Thereafter, a small amount of that venom is used to immunize the production animals (e.g., horses or sheep). After the animals have built up sufficient immunity (high plasma titers of antibodies) against the target venom, the blood plasma is extracted from the animals, and the immunoglobulin G antibodies are purified by various protein precipitation techniques. Finally, the antibodies are formulated and bottled for human use.

Most envenomings and some poisonings (e.g., botulism) are currently treated with serotherapy [36,37]. Serotherapy is based on some of the same principles as vaccination, which was first developed by Edward Jenner [38]. However, instead of inducing immunity in the patient directly, immunity is induced in a production animal and the hyper-immunized serum is transfused into the patient, also known as passive vaccination (Figure 3) [36]. A key advantage of this technique is that in the case of highly diverse toxin cocktails that are commonly found in venoms, it is not essential to know what specific toxins are present, as long as the immune system of the production animal gives rise to neutralizing antibodies against all the key toxic components [39]. This approach has proven very effective over the last century and has saved countless lives. However, conventional serotherapy suffers from many drawbacks. Venom extraction from animals involves a significant danger to the personnel handling the venomous animals. Another key issue is the use of production animals, which is costly, since keeping production animals and ensuring their well-being is expensive [40,41]. Immunization of animals is also a time consuming process with inherent batch-to-batch variation, which can have considerable therapeutic implications [42,43,44]. The presence of non-toxic immunogens in the venoms used for immunization is likely to decrease the concentration of therapeutically relevant antibodies in an antivenom, as these components may give rise to irrelevant antibodies of no or low therapeutic value [12]. In fact, a study on equine scorpion antivenoms demonstrated that only a small percentage of the antibodies present in the antivenom were able to neutralize important venom toxins [45]. Raising a significant titer of antibodies against small venom components with low immunogenicity and high toxicity has been demonstrated to be particularly problematic [46,47,48,49]. Furthermore, the use of non-human antibodies results in a high risk of both early and late adverse reactions [50,51], such as serum sickness and anaphylactic shock, since these antibodies are immunogenic due to their heterologous nature. Finally, due to the very minute amounts of venom that can be extracted from many venomous animals (especially spiders and scorpions), production of antisera is dependent on laborious venom collection processes, where large numbers of animals need to be milked by electrostimulation in order to procure enough venom for immunization. Such challenges necessitate significant technological innovation for the production of safer and more effective antivenoms, as well as an increase in the economic sustainability of the production process itself, by making it independent of both venoms and animals [50,52].

## 4. Human Monoclonal Antibodies

Monoclonal antibodies (predominantly IgGs) are highly specific for their target, and since many toxins are structurally distinct from the proteins present in the human body [53,54], few adverse off-target effects can be expected when using mAbs to target these. The nature of mAbs provides inherent advantages over serotherapy in the context of neutralizing toxins. With a well-defined mAb preparation, where specificity is predefined and only one immunoglobulin isotype is present, industrial production can be performed with a low batch-to-batch variation [54]. Additionally, the presence of only specific antibodies ensures high biological activity per mass of protein [55], lowering both the cost of treatment, as less material is needed, and the risk of late onset adverse effects [12].

Due to the protein size, IgG-based antivenoms have low volumes of distribution, cycle through the interstitial space many times, and have long elimination half-lives [56,57]. Importantly though, the long elimination half-life is especially a result of binding to the neonatal Fc receptor, which reduces lysosomal degradation and recycles the IgGs [58,59,60,61]. Human IgGs have an elimination half-life of 21–28 days [58,59,60,61], chimeric IgGs a half-life of 8–10 days, and murine IgGs 1–3 days [60,62]. For these reasons, full length IgGs have the potential of effectively neutralizing systemically acting toxins in the intravascular compartment for many days [12]. Besides the neutralizing ability of their variable regions, IgGs have an Fc region that mediates opsonization and activation of the complement system [63]. Conversely, antibody formats without the Fc region can be exploited to obtain a larger distribution volume and faster tissue penetration [58,64,65,66], though at the cost of a much shorter half-life of 0.5–30 h [60]. Such formats can be used for neutralizing systemically acting toxins as well as locally acting toxins. The advantages and disadvantages of various antibody formats for antivenom development have been thoroughly reviewed elsewhere [12].

Non-human mAbs are immunogenic and can elicit an immune response when administered to human recipients [67]. With humanized or human mAbs on the other hand, the adverse reactions from introducing foreign antibodies in the human body are considerably lowered [54], and several studies have investigated the use of human mAbs for toxin neutralization. Human mAbs capable of neutralizing the hemorrhagic metalloproteinase HR1a from *Protobothrops flavoviridis* have been developed by Morine et al. and used to map epitope regions on the HR1a toxin [68]. Additionally, the use of human mAbs has been investigated for the neutralization of shiga toxin [69], *Clostridium difficile* toxins [70], Staphylococcal enterotoxin [71], ricin toxin [72], anthrax lethal factor [73], and botulinum toxin [74]. Most recently, a study for the very first time demonstrated the use of fully human mAbs to neutralize animal toxins in vivo. Additionally, it highlighted the potential of oligoclonal mixtures of recombinantly expressed fully human mAbs in treatment of envenoming, by presenting their capability of neutralizing experimental snakebite envenoming [18].

Cost-competitive production of antivenom antibody mixtures affordable even in poor regions of the developing world is a major challenge [75], but with the rapid growth in clinical use of mAbs [76,77] it seems possible to achieve in the future. Currently, expression systems based on Chinese Hamster Ovary cells are the most common choice for the industrial manufacturing of recombinant monoclonal antibodies [76,77], although microbial expression is also being explored for the production of various antibody formats [12]. Mammalian cell lines are preferred for the expression of IgG molecules [76,77], as they enable post-translational glycosylation, and the generation of antibodies with low immunogenicity, whilst also ensuring the proper folding and secretion of large proteins. Ultimately, a high yield of functional proteins can be obtained [78,79], and often the industrial production of IgG yields more than 12 g/L [79]. However, mammalian expression systems require expensive media, and the cost for disposables and other consumables is typically high [79]. While prokaryotic expression systems in many cases may be used for low-cost manufacture of simpler proteins, these systems are not yet capable of correctly glycosylating antibodies. Adding to this, the disulfide bonds of antibodies can usually not be obtained in the reducing environment of the bacterial cytoplasm, wherein antibodies also tend to fold incorrectly and form insoluble aggregates ultimately leading to lower expression yields [12,80]. Alternative binding proteins with characteristics such as small size, stable structure, and lack of disulfide bonds and glycosylation sites might be attractive in order to properly exploit the simple and cheap prokaryotic expression systems and obtain advantages such as large volume of distribution and rapid tissue penetration.

## 5. Alternative Binding Scaffolds

Alternative binding scaffolds offer potential improvements to both the cost and efficacy of antitoxin therapy versus traditional serotherapy, and even monoclonal antibody formats. Improvements to cost can be split into three areas (i) facile engineerability to allow for a cheap and rapid research and development phase, (ii) low production costs at good manufacturing practice (GMP) quality, and (iii) high stability at elevated temperatures with a low propensity for aggregation to reduce the need for, and the associated cost of, a cold-chain and storage facilities.

Facile engineerability of a scaffold can be achieved by compatibility with well-established binder discovery and development techniques, such as phage display, ribosome display, or yeast display. The libraries that are screened using these display techniques should be of high quality i.e., containing as diverse a set of potentially functional variants as possible. Knowledge of the binding interface of a scaffold is useful so that relevant residues/regions can be diversified to alter target binding without creating a large percentage of inactive variants. Further development and engineering are also greatly facilitated if the intended final drug format is the one used in the initial discovery stage. Of note here is the process of IgG antibody discovery, in which phage display of Single-chain variable fragment (scFv) or Fragment antigen-binding (Fab) molecules is often used, even though the intended final drug format is often full IgG. Conversion of a binder in the scFv format to an IgG format may not be trivial with a loss of affinity and activity often being experienced [81,82,83]. In contrast, the discovery processes for all the herein discussed alternative binding scaffolds would use the same molecular format throughout.

Once a desired antitoxin has been developed, it is necessary to produce it in a monoclonal, biochemically defined manner, whilst also maintaining low costs at GMP quality. To achieve this goal, it is desirable for the scaffold to have no requirements for post-translational modification (e.g., glycosylation, or formation of disulfide bonds), to consist of only a single domain, and to be expressible in high yield without aggregation in microbial (bacterial or eukaryotic) platforms. Alongside recombinant expression, it is also worth noting that some of the smaller scaffolds have already been demonstrated to be compatible with chemical synthesis [84]. Whilst the effect of chemical synthesis on cost is difficult to predict, its use does allow for the incorporation of moieties that could provide useful biochemical properties (e.g., D-amino acids for resistance to protease activity) [85].

High-yield recombinant expression is often coupled to a scaffold being highly stable and soluble. These two characteristics are also fundamental considerations for reducing the cost of future antitoxins as they could enable therapies to be delivered and stored in resource-poor or remote environments without the need for a cold chain. In turn, this may potentially allow for such antitoxins to be used as first-aid treatments, as they could be stable at elevated temperatures in the field setting. Many alternative scaffolds exhibit higher thermal stability than current antibody formats, with some even being able to withstand extreme temperatures whilst maintaining functionality (DARPins and affimers). Data on long-term storage is difficult to attain; however, a notable example does exist with DARPins that in one case have been shown to retain 97.6% monomeric status in phosphate-buffered saline at 15 mg/mL after 6 months at 25 °C [86].

The use of alternative scaffolds as antitoxins also has the potential to improve both the efficacy and safety of envenoming therapy. This is notably in relation to immunogenicity, tissue penetration, and percentage of therapeutically active components, which can all also affect the cost of therapy due to a requirement for repeated dosing if not optimized (reviewed by [9]). Bearing this in mind, one important method for improving safety is to minimize the potential immunogenicity of antitoxins. As such, the use of a scaffold derived from human proteins is attractive because the chance of a patient’s immune system recognizing the protein as foreign is reduced. To confirm this low immunogenicity, it is beneficial to see that a scaffold has already been used in the clinical setting and is well-tolerated in humans over a wide-range of doses in numerous clinical trials. The non-antibody scaffolds that have so far undergone most testing are nanobodies and DARPins, for which selected molecules are currently in Phase 3 clinical trials, and the approved Kunitz domain drug Ecallantide [25]. Successful clinical trials also demonstrate that there are no, or minimal, innate scaffold-specific off-target or undesired interactions that would jeopardize the safety of a patient. However, the majority of worries surrounding off-target effects would need to be evaluated on a case-by-case basis, as off-target toxic interactions would most likely be a characteristic of the individual binding mechanism of the investigated variant of a scaffold, rather than a general characteristic of the scaffold.

To improve both the safety and reduce the cost of therapy it is desirable to administer the minimum possible dose of total protein to a patient. This can be achieved by maximizing the percentage of therapeutically active components in an antivenom. Current approaches (e.g., phage display) of discovering binders in a monoclonal manner against desired targets enables a rationally defined mixture of binders to be used in the final therapeutic composition, ensuring a maximal percentage of active components. All herein discussed scaffolds are compatible with this monoclonal discovery process. A high affinity interaction engineered/evolved to be significantly better than current serotherapy affinities could allow for a lower dose of total protein to be administered to the patient, due to the high percentage of therapeutic content. Thus, scaffolds exhibiting this ability are highly desirable. As examples, DARPins and nanobodies have been developed to have affinities in the low picomolar to femtomolar range [87].

The most notable area in which alternative scaffolds can potentially offer improvements to current antitoxin efficacy is tissue penetration. All scaffolds to be discussed are significantly smaller than current IgG or Fab based serotherapy molecules (e.g., bicyclic peptides can be 100-times smaller than IgG molecules), thus enabling increased tissue penetration and potential improvements to the efficacy of toxin neutralization [88]. Increased tissue penetration can be a particularly relevant characteristic for neutralizing toxins that act locally, such as snake venom metalloproteinases and myotoxic phospholipases A_2_ (PLA_2_s) [2]. However, the smaller size of many of the non-antibody scaffolds places these scaffolds below the glomerular filtration limit (~70 kDa), causing the molecules to be rapidly removed from circulation unless otherwise modified [89]. Many successful attempts have already been made to modify several of the different scaffolds to increase their half-lives involving techniques such as PEGylation (attachment of polyethylene glycol chains), fusion to Human Serum Albumin (HSA), fusion to the Fc domain of IgGs, or creating a bispecific binder against a target and HSA or Fc receptors [90,91,92,93,94]. These modifications have dramatically improved the half-lives of associated proteins from minutes to days. It is worth noting that these improvements are not solely due to an increase in size, but also address another aspect lacking from non-antibody scaffolds; the lack of an interaction with the immune system via Fc receptors. Cell-mediated effector functions are not required for toxin neutralization; however, these scaffolds do lack an important interaction with the neonatal Fc receptor. This intracellular Fc receptor is responsible for recycling pinocytosed IgGs and serum albumin back to the bloodstream. Proteins in the bloodstream that do not possess this interaction are instead subjected to lysosomal degradation in the cells, contributing significantly to the low half-lives exhibited by the unmodified scaffolds [95]. Thus, fusion to the Fc domain, HSA, or a binder against either of these molecules or the Fc receptor itself, addresses this issue.

Alternative scaffolds could also enable entirely new efficacies, compared to full IgG antibodies, to be achieved due to the use of different methods and sterics of binding to a target. Toxins represent an extremely diverse field of targets, ranging from small molecules that exert their function via a binding interaction with a target macromolecule, to enzymes that exert their toxic function by catalyzing a reaction. Whilst neutralizing a toxin that interacts with macromolecules could be achieved by blocking a surface localized interaction site or altering the toxicokinetics, the specific neutralization of an enzyme requires binding to an allosteric regulatory site, blocking access to the substrate cleft, or interaction with a buried active site. Interestingly, heavy-chain antibodies from camels have already exhibited an increased enzyme-inhibitory profile compared to full IgGs, likely due to the ability of the long Complementarity-Determining Region 3 (CDR3) loop of their heavy chains to stretch through a narrow substrate channel before reaching an active site [96]. These channels can be very long on a molecular level, with 64% of enzymes having a channel greater than 15 angstrom in length, with the typical being 28 angstrom [97]. An IgG has a diameter of 200-400 angstrom; thus, it cannot fit into a substrate channel [98]. Small molecules would traditionally be the entities of choice for enzyme inhibition (such as the PLA_2_ inhibitor Varespladib), however, protein scaffolds could also be of use [99]. Notably, the discovery of protein-based enzyme inhibitors could be simpler than small molecules due to the ability to leverage the power of ultra-high throughput directed evolution for drug discovery. The scaffolds used should either be extremely small, for instance bicyclic peptides, or possess a binding interface with a long protuberance from the scaffold as is the case with nanobodies or LoopDARPins [100].

All of these characteristics will subsequently be discussed and evaluated on an individual scaffold level.

### 5.1. Nanobodies

The nanobody technology was developed after the discovery that Camelidae (e.g., camels and llamas) possess fully functional antibodies that only consist of heavy chains [101,102]. These heavy-chain only antibodies encompass two constant domains (C_H_2, C_H_3), and a single variable domain (V_H_H), which have antigen binding capacity comparable to human IgGs and have proven to be very stable. The single-domain antibody consists of 110–136 amino acids, comprising one variable domain (V_H_) of a heavy-chain antibody. These single variable domains, with their small size and unique structural and functional properties, form the base of a cohort of therapeutic molecules, which are known as nanobodies (Nbs; Figure 1(1)) [103]. There have been multiple reviews covering Nbs in depth [104,105,106], hence, in the following, only key features are briefly introduced.

Nbs are known to possess high affinity and specificity (similar to whole antibodies), good solubility (20 mg/mL), high thermostability (T_m_ up to 86 °C), higher penetration rate into deep-tissue due to their small size (12–15 kDa), and low production cost [104,107,108,109,110]. Due to their small size and their extended CDR3 loop, Nbs prove to be adept at neutralizing targets via binding hidden epitopes that are not accessible to IgGs [111], such as the active sites of enzymes, intracellular targets, G-protein coupled receptors, and ion channels [105,112,113]. Compared to mAbs, Nbs possess a different pharmacokinetic behaviour owing to their relatively short half-life (few hours) [111], which can be advantageous in applications where rapid clearance is required, but which can also be a disadvantage when targeting animal toxins that have the possibility to reside in a bite wound for a longer period of time. The half-life can, however, be improved by PEGylation or conjugation to HSA [90], consequently sacrificing its small size properties and possibly resulting in less tissue penetration. Nonetheless, Nbs are considered to have very low immunogenicity, because of their homology with human V_H_ sequences, and can also be humanised by grafting the CDR loops onto a human V_H_ scaffold. Low immunogenicity and efficacy has led to a significant interest in this scaffold for research, diagnostic, and therapeutic purposes [114]. Over a dozen clinical trials have been carried out using Nbs in multiple areas of therapy, with only two being terminated prematurely due to adverse effects [106]. It should be noted that these adverse effects may have been target biology related rather than scaffold-related as the effects were disease related, e.g., when targeting the AB peptide in Alzheimer’s disease the peptide’s concentration was seen to increase rather than decrease over time [106]. Due to their good tissue distribution, Nbs are likely to more easily reach and neutralize toxins in distal tissues compared to mAbs, thus potentially being capable of neutralizing toxins closer to the depot of the bite wound, responsible for recurrent symptoms after some snakebites [115].

Phage display is the first choice when it comes to discovery of Nbs due to its robustness [104,107,108,109,110]. However, other display techniques have also been employed, such as ribosome or mRNA display, bacterial or yeast surface display, as well as bacterial two-hybrid screening [115]. In 2013, Richard et al. successfully isolated high affinity llama V_H_H’s against the α-cobratoxin (α-Cbtx) from *Naja kaouthia*. Futhermore, they were able to completely neutralize the lethal effects of the α-Cbtx (with a ratio of less than one Nb per toxin molecule), and later on enhance the thermal stability of the discovered V_H_H’s, by introducing a mutation and a disulfide bridge [116,117]. Nb antitoxins have also successfully been discovered against toxin fractions from the desert scorpion, *Androctonus australis hector*, which showed an advantageous balance between toxin neutralization capacity and fast renal clearance, resulting in low liver uptake of the nanobody [118,119,120].

### 5.2. Affimers

Phytocystatins are small protein inhibitors of cysteine proteases and have been the inspiration for designing an artificial protein binding scaffold termed adhiron (Figure 1(2)). By generating a plant-derived consensus phytocystatin protein, Tiede et al., 2014 established a basis from which the artificial adhiron constructs could be derived [121]. The cystatin structure of adhirons resembles that of an earlier scaffold based on stefin A [122], and both scaffolds are now collectively referred to as affimers [123].

The artificial affimer proteins are characterized by a four-stranded antiparallel β-sheet core and a central α-helix. This is a compact structure with a melting temperature (T_m_) of 101 °C [121], which renders it more stable than traditional antibodies [121]. With a small size of around 100 amino acids (~11 kDa), a high solubility, and a high stability [121], affimers exhibit rapid tissue penetration and rapid target retention [124]. Furthermore, these small monomeric proteins can be easily multimerized or fused with other scaffolds to obtain multispecificity or improved pharmacokinetics [124]. No disulfide bonds are present in affimers [121], enabling them to be properly expressed in reducing intracellular environments. They also do not contain glycosylation sites that require post-translational modifications, suggesting that microbial expression should be achievable on an industrial scale [121,124]. Indeed, with *E. coli* as host, purification yields of soluble affimer at 10–100 mg/L can be obtained in an experimental setting, if a heating step is included [121].

Affimer libraries are generated by replacing four amino acids between the first and second β-strand and three amino acids between the third and fourth β-strand, with two loops consisting of nine random amino acids each. The insertion of peptide sequences for molecular recognition in these loop positions results in libraries with flexible and extended binding regions. These regions are expected to adapt to a conformation that enables molecular contact with a wide variety of targets, which in turn enables affimers to interact with protein pockets and surfaces as well as peptides and small molecules [121]. Affimers selected through phage display have displayed affinities in the nanomolar range [121]. Also, like that of their phytocystatin parent proteins, affimers have been developed to have high activity as protease inhibitors [121]. Affimers have successfully been selected against 350 different targets, including proteins, peptides, organic molecules, and inorganic metallic nanoparticles [125]. The in vitro discovery platform enables selection against targets that are hard to raise antibodies against, either due to their low immunogenicity or high toxicity [125], and circumvents the issue of having to humanize heterologous IgGs.

Recent studies have demonstrated the ability of affimers to inhibit protein–protein interactions [126,127]. While one study demonstrated inhibition of the interaction between the IgG immune complex and the Fc gamma receptor FcγRIIIa [121,128], another study isolated affimers inhibiting small ubiquitin-related modifier-dependent protein–protein interactions with isoform-specificity [121,128]. It remains to be fully investigated whether affimers are suitable for therapeutic purposes, as so far only pre-clinical studies have been undertaken with a PD-L1 inhibitor, but the properties of the scaffold are attractive [121,128].

### 5.3. Adnectins (Monobodies)

Adnectins are based on the tenth fibronectin type III domain (^10^Fn3), which functions as an integrin binder in humans (Figure 1(3)) [129,130]. The adnectin family constitutes one of the earliest designed binding proteins, and the scaffold design was instigated by the first constructions of libraries based on ^10^Fn3 starting in 1998 [129,131,132]. The interest in ^10^Fn3 was sparked by its structural similarity to the variable domains of antibodies, its biophysical properties, and its abundance in human blood and extracellular matrices [129].

Adnectins are small and compact artificial proteins with a molecular size of ≤12 kDa [129,133]. The structure is composed of seven β-strands joined by six loops, forming a two antiparallel β-sheet fold, where the loops at both poles are accessible for solvents. The monomeric adnectin structure is ideal for multimerization, where multi-functional binding proteins can be obtained [129]. The diversifiable loop regions are very similar to the variable domains of antibodies, but the protein sequence is not homologous to that of immunoglobulins. Adnectins further set themselves apart from antibodies by not containing any disulfide bonds or free cysteines [129,130], by exhibiting a high T_m_ of up to 84 °C [134,135,136], and by their ability to retain the high thermostability under reducing conditions, enabling high protein yields in bacteria [129]. Additionally, adnectins are not glycosylated [91], which further enhances the ease of cost-efficient production in a bacterial expression system.

The three loops at one pole in ^10^Fn3 are structural analogues of the H1, H2, and H3 CDRs of antibodies, and are of highest interest when generating artificially diversified surfaces for target-binding in adnectin libraries [137,138]. Diversification might result in lower thermostability and solubility, but the very high stability of the wild type scaffold ensures that even destabilized variants retain sufficient stability to be exploited therapeutically [133,139,140,141]. When the scaffold structure is retained, and variations in sequence and length are only introduced in the variable loop regions, structural stability and binding affinities in the sub-nanomolar range can be obtained [91]. Their small size, soluble nature, and great stability already suggests a high volume of distribution and rapid tissue penetration, but as their small size will also result in a rapid clearance by the kidneys [91], ensuring prolonged half-life by improved pharmacokinetics might be of relevance for their use in envenoming therapy.

The therapeutic potential of adnectins remains unstudied for toxin neutralizing purposes, but at least three separate molecules have been investigated in clinical trials up to Phase II for other indications, e.g., in oncology [25,129]. Additionally, some studies have investigated their ability to bind soluble proteins. One target is the protein convertase subtilisin/kexin type 9 (PCSK9) enzyme that is commonly targeted to decrease low density lipoprotein (LDL) in cardiovascular disease. Adnectins binding the PCSK9 enzyme have successfully been discovered with sub-nanomolar affinity to sterically prevent interactions with LDL receptors [91]. Furthermore, PEGylation of the adnectins did not hamper this competitive inhibition [91]. Another study describes the generation of an adnectin that was capable of binding immobilized interleukin 23 (IL-23) with a dissociation constant (K_d_) of 2 nM, and which inhibited IL-23 from binding its receptor. Furthermore, the adnectin demonstrated a half maximal inhibitory concentration (IC_50_) of 1 nM in a biochemical competition assay with the IL-23 receptor [130].

### 5.4. Affibodies

Affibodies are a type of protein scaffold that was first developed in 1997 and is based on the Fc-binding B domain of the staphylococcal protein A (SPA; Figure 1(4)) [142]. An affibody consists of a single engineered Z domain, which is a 58 amino acid residue variant (~6 kDa) of a consensus SPA B domain, forming a three-α-helix-bundle structure [142] with a T_m_ of 75 °C [143]. Affibodies with high affinity to their target proteins are selected using phage display from combinatorial libraries, where 13 surface-located residues on helix 1 and 2 have been randomized [142]. Affibodies have shown affinities in the range from micromolar to low picomolar dissociation constants and can be improved by affinity maturation [144,145].

One major challenge to overcome for affibodies is rapid renal clearance. One strategy employed to prolong serum half-life and impede renal excretion has been to fuse affibodies to an HSA-binding domain [144].

The affibody scaffold has been widely applied within bioseparation, diagnostics, functional inhibition, viral targeting, and in vivo tumor imaging/targeting [144]. Affibodies have been used as target-specific probes that bind with high affinity to several cancer-associated targets. Preclinical and clinical studies have shown that affibodies are devoid of toxicity and immunogenicity [146], indicating that their non-mammalian origin might not be of particular concern. However, when one of the affibodies was fused was fused with a radiometal, the renal accumulation clearly exceeded that of the tumor, hindering safe therapeutic applications.

### 5.5. Affitin (Nanofitins)

Affitins (commercial name Nanofitins) are artificial proteins able to selectively bind antigens (Figure 1(5)). They originate from the DNA binding protein 7d (Sac7d) found in various Archaea, such as *Sulfolobus*, *Acidianus*, and *Metallosphaera* genera. Sac7d consists of one protein chain of ~66 amino acids (~7 kDa) folded in an oligonucleotide/oligosaccharide-binding-fold, which is formed by a β-barrel capped by a C-terminal α-helix, lacking disulfide bridges [147].

Affitins were discovered and developed by Mouratou et al. in 2007, yielding small and stable intracellular inhibitors with no disulfide bridges and the ability to be produced at high levels in *E. coli* [148]. Their affinity can be engineered as desired through randomization of amino acids on the binding surface and subjecting the resulting protein library to ribosome display selection [148]. Having already been used as specific inhibitors for enzymes, it is possible that affinity can be directed towards a wide range of targets, including peptides, proteins, viruses, and bacteria [149,150].

Affitins are extremely heat resistant (up to 90 °C) [151], originating from a thermophile organism, and can have up to 18 of their amino acids mutated (27% of total), providing them with tolerance towards several randomization schemes, while conserving both their fold and advantageous properties [150]. Additionally, affitins have the potential to cover and/or deeply penetrate active sites [149]. In order to further improve on the properties of affitins, Aho7c originating from *Acidianus hospitalis*, possessing picomolar affinities (K_d_ of 110 pM), high stability (up to 74 °C; pH 0–12), and a 10% smaller size (60 compared to 66 amino acids) has been characterized [152]. However, despite the constant development of the small-size affitins, they have yet to undergo significant clinical testing to evaluate their safety and efficacy.

### 5.6. Anticalins

Anticalins are single polypeptides of 150–180 residues, artificially engineered from lipocalins to bind ligands with high affinity in a deep complementary pocket, resembling that of the antigen-binding site of antibodies [153,154,155], and were first described in 1999 (Figure 1(6)) [153,154,155].

Since initial discovery [156], lipocalins have been found to exist in many organisms with more than 15 isotypes found in humans alone [154]. As small, secreted proteins of less than 20 kDa, lipocalins often function as transport or storage proteins for hydrophobic and/or chemically sensitive organic compounds, e.g., vitamins, lipids, and steroids. Lipocalins share a structurally highly conserved β-barrel composed of eight antiparallel β-strands around a central core. Four structurally variable loops form the entrance to the ligand-binding pocket that resembles the hypervariable region that forms the antigen binding site of antibodies [155].

Lipocalins are endogenous human plasma proteins [157] and are rather stable with T_m_ reaching almost 79 °C [158]. Anticalins engineered from lipocalins have affinities in a range comparable to antibodies and, when optimized, K_d_ values of approximately 1 nM and 800 pM have been achieved for fluorescein and digoxin, respectively [155]. Additionally, anticalins have the advantages associated with small sized proteins and simple molecular structure with four variable loops that are less complex than the CDRs of antibodies and thus require less manipulation [157]. To identify ligand binders, anticalin libraries are created with targeted random mutagenesis of 16–24 amino acids at exposed positions, which is primarily the tips of the four loops [159]. Subsequently, phage display selection and microculture screening in combination with ELISA is used for identification of lead anticalin candidates [157]. To prolong the inherently short plasma half-life or to generate multispecific fusion proteins offering novel therapeutic modalities, a free cysteine residue or the N or C-terminal can be used for site-specific covalent attachment. Anticalins do not have constant Fc regions, for which reason undesired immunological effector functions may be avoided [155,157]. They can be effectively produced in microbial expression systems, e.g., *E. coli*, as many naturally lack glycosylation and can be engineered to be free of the one or two disulfide bonds they often contain. [155,157].

Currently, anticalin-based biopharmaceuticals for the areas of oncology and inflammation are in development by the company Pieris [157]. To date, five drug candidates have passed early clinical development, where they have demonstrated tolerability and stability. Two drug candidates are in Phase I clinical trials, three have completed Phase I clinical trials, and one of these is now in Phase II clinical trial. All of these drug candidates are based on human lipocalin scaffolds in order to reduce the risk of immunogenicity in patients upon repeated dosing [157]. One example is the PRS-050 anticalin protein that targets and tightly binds vascular endothelial growth factor A (VEGF-A) with a K_d_ ~20 pM, thereby preventing receptor binding and subsequent activation. VEGF-A is a key factor in tumor-initiated angiogenesis and ocular diseases. To extend the plasma half-life in vivo, the anticalin was engineered with site-directed PEGylation [92].

Another example is the anticalin PRS-080, which targets hepcidin that plays a major role in iron metabolism, especially for patients with functional iron deficiency anemia, as hepcidin blocks iron export from the storage cells in the body. Prolonged circulation is achieved by site-specific PEGylation by addition of a 30 kDa PEG polymer [157]. Furthermore, anticalins have shown promising short-term efficacy as an antidote against digoxin intoxication in rats [160].

### 5.7. Armadillo Repeat Proteins

In 1980, Nüsslein-Volhard and Wieschaus reported the development of a line of *Drosophila melanogaster* that had an altered segmental patterning, depending on specific mutations [161]. By 1989, Riggleman et al. reported that mutations in the *armadillo (arm)* gene conferred this segment polarity [162], which later was discovered to be encoding β-catenin. β-catenin was shown to contain what was then called an armadillo repeat (ArmR), and proteins containing these were later found in many eukaryotes, where they mediate signaling, nuclear transport, and cell adhesion (reviewed in Tewari et al. 2010 [163]). These naturally occurring armadillo repeats proteins are termed nArmRPs, in contrast to designed dArmRPs.

An ArmR domain consists of 42 amino acids with an approximate molecular mass of 4.6 kDa, which compose three helices (H1, H2, and H3; Figure 1(7)). These domains stack together in repeats of 4-12, creating ArmRPs having the structure of a right-handed superhelix [164]. As the ArmRs stack together, their H3 motifs form a concave binding pocket, containing an interaction site that can bind a target in an extended confirmation, meaning for instance an unstructured peptide region [164,165]. Importantly, the target extended peptide surface has to have certain negatively or positively charged residues (depending on ArmRP subfamily) in order for the ArmRP to bind [164,166]. Binding proceeds in an antiparallel fashion in regard to the ArmRP, resulting in an asymmetric double helix [164].

The stacked motifs have been reported to achieve nanomolar dissociation constants, indicating strong binding to their interaction partner. ArmRPs have conserved residues in the hydrophobic core of the protein, conferring stability [164]. Furthermore, conserved asparagine residues contribute to protein-protein interactions together with other residues at the binding interface, which can be engineered to achieve specificity [164,166,167]. In the final ArmRP library, a diversity greater than 10^11^ was achieved, of which almost all were stable [164,168]. In this final library, and after significant engineering efforts [164,165,169,170], six randomization positions per repeat could be achieved, conferring a theoretical diversity of 9.9 × 10^6^ per repeat [168], and by stacking of the repeats, target specificity is obtained. As target specificity was obtained by stacking of this modular system, the system has the potential to allow for generation of preselected repeats for certain short pieces of target peptide that can be custom designed and assembled on demand into a new protein that can bind a prescribed, extended peptide [171].

However, as only peptides without conformation can be targeted by the ArmRPs, the proteins may have limitations in therapeutic applications. Additionally, off-target effects may merit pre-evaluation of risk through a homology scan of native, endogenous peptides. Conversely, mutations in nArmRPs (or ArmR-containing proteins) have been linked to a number of diseases, such as Parkinson’s disease [172], neuroblastoma progression [173], and Bilateral macronodular adrenal hyperplasia [174]. Also, designing preselected repeats may have the drawback that significant deviations occur in the ArmRP’s curvature upon changes in environment, such as specific solutions, making it more difficult to design for larger target peptides [171]. Another obstacle is the expected rapid clearance from the circulatory system, which may prevent the ArmRPs to efficiently exert their effects, although it is yet to be tested in vivo. However, as with other small size molecules, this might be possible to mitigate by conjugation or fusion to a targeting domain, or by fusion to a carrier protein.

dArmRPs have thus far been reported to recognize alternating lysine and arginine residues with affinities in the picomolar range [169]. No clinical trials have been reported, making evaluation of potential immunogenicity or toxicity difficult.

### 5.8. Avimers

In 2005, Silverman et al. developed a new class of binding proteins called avimers, short for ‘avidity multimers’ (Figure 1(8)) [175]. These proteins were developed based on a large family of 217 known A-domains in human extracellular receptors [175].

Each A domain is ~35 amino acids (~4 kDa) and has 6 conserved cysteines, forming three disulfide bridges, and also 6 conserved acidic residues that coordinate calcium binding, together conferring structural integrity [176]. This leaves ~30 non-conserved residues per A-domain that can be designed by exon shuffling to achieve specificity to a desired target molecule and selected by phage display [93,175]. Furthermore, connecting A-domains by linkers led to the ability of designing multimers (avimers) that achieve avidity by the individual A-domains’ summed affinity to different epitopes in the target molecule, or cross-reactivity by linkage of A-domains with different specificities [175].

Avimers have been reported to have high thermostability and will still be active after 2 weeks incubation time at −80 °C to 50 °C, and after 5 days at 90% human serum at 37 °C [93,175]. Multimeric A-domains of up to 8-mers have been expressed in bacterial high-density fermentation production, with yields of more than 1.4 g/L [93,175].

Two to three-mer avimers have been reported to obtain an avidity in the sub-nanomolar range, binding strongly to their target molecules [175]. As avimer libraries have been based only on amino acids at the various positions from naturally occurring human A-domains, there is a bias towards hydrophilic residues, which contribute to solubility at concentrations of greater than 70 mg/mL [175].

In terms of application, in 2018, Hulme et al. [177] used phage display selection to identify A domains with subnanomolar avidity and specificity to type II collagen. Fusion of the avimer to anti-IL-1Ra ensured access to the extracellular matrix, whilst tethering the therapeutic close to its target (IL-1 receptor). This extended the residence time, and thus increased the therapeutic efficiency, while no immunogenicity was reported. Silverman et al. demonstrated in 2005 the ability of the tetrameric avimer, C326 (AMG220), to specifically bind IL-6 with picomolar affinity, thus preventing IL-6 mediated inflammation [175]. The avimer consisted of fused A-domains with specificity to IgG, which prolonged serum half-life, and three other domains with specificity to IL-6, together providing the protein with high avidity. C326 (AMG220) did enter phase I clinical trials in Australia but the study has been put on hold (clinical trial ID: NCT00353756).

### 5.9. β-Hairpin Mimetics

β-hairpin mimetics consist of a single β-hairpin motif, i.e., two antiparallel β-strands that are connected by a loop (Figure 1(9)). These motifs are frequently found at protein-protein interaction interfaces, and thus, by mimicking these motifs, one can inhibit or activate a desired molecular target. By designing a synthetic β-hairpin to replicate native protein epitopes, Fasan et al. demonstrated the ability to mimic the HDM2 binding loop of p53, as it binds to HDM2. When this interaction occurs, it can further activate tumor suppressor genes in cancerous cells, as p53 is a tumor suppressor protein [178,179]. This β-hairpin was 15 amino acids long (1.8 kDa), containing an 8-residue variable loop, preorganized by d-Pro-l-Pro [180], which achieved an IC_50_ to HDM2 of 1.1 µM.

In 2013, Karpova et al. demonstrated the use of a protein epitope mimetic (PEM) for antagonizing C-X-C chemokine receptor type 4 to mobilize hematopoietic stem cells for therapy by direct cellular targeting [181]. The PEM, POL5551, was well tolerated up to 100 mg/kg in mice studies, indicating low toxicity, which may thus be the case for other PEMs as well [181]. POL5551 exhibited a clearance of more than 90% after 4 h, but has yet to be tested in clinical trials. Therefore, off-target effects and immunogenicity remain unknown, but due to the molecular size of the mimetics, rapid clearance would also be expected for other PEMs. This could, however, be dealt with by fusion to a carrier protein, or by linkage to a targeting-protein, in order to bind a serum protein for increased half-life. It is also worth noting that this scaffold is compatible with chemical synthesis [182].

### 5.10. Bicyclic peptides

Polycyclic peptides are naturally occurring (e.g., in soil bacteria, venom from cone snail, etc.), however, it is only since 2009 that the bicyclic peptides have been investigated in a therapeutic context (Figure 1(10)) [183,184,185]. Bicyclic peptides are highly constrained and approximately 2 kDa (9 to 15 amino acids) in size [186]. These peptides are highly soluble and provide manufacturing and formulation flexibility. In comparison to their monocyclic counterparts, the bicyclic peptides harbor increased conformational rigidity due to their two rings, rendering each ring smaller, and thus providing increased stability. This extra rigidity can result in molecules with high target specificity and affinity [183]. Because of their simplicity, they can be chemically synthesized, significantly lowering the cost of manufacture [183].

Owing to their properties, bicyclic peptides have proven to be a promising candidate for therapeutic applications. They can be engineered to serve as therapeutics against a diverse set of targets, and their proteolytic stability with improved plasma stability (relative to linear peptides) makes them highly relevant for targeting soluble antigens. This high proteolytic stability is exemplified by a bicyclic peptide against streptavidin that retained 96% of its activity even after 24 h of incubation with chymotrypsin [187]. However, their small size does result in rapid clearance from the kidneys [183].

Bicyclic peptides have only recently been investigated as drug candidates by the company Bicycle Therapeutics. Bicycle Therapeutics are focusing on Bicycle Drug Conjugates, a format in which a potent toxin is conjugated to a bicyclic peptide designed to bind tumor antigens, thus mediating targeted cytotoxicity. Notably, the conjugation of toxin to bicyclic peptide is via a tumor microenvironment specific cleavable linker. The linker and coupling chemistry hold the attached toxin inert until the conjugate is localized within the tumor microenvironment. This strategy limits the body’s exposure to the conjugated toxin, reducing the risk of potential damage in normal tissues. Their lead compound, BT1718, currently in phase I/IIa, targets MT1, which is an antigen present in many solid tumors, including breast, lung, ovarian, and colon cancer, responsible for breaking down the proteins usually surrounding the cell, allowing cancer to grow and spread [188].

### 5.11. DARPins

Designed Ankyrin Repeat Proteins (DARPins) are an artificial protein scaffold based on Ankyrin Repeat (AR) proteins, which mediate diverse protein-protein interactions in nearly all species (Figure 1(11)) [189,190]. The majority of natural AR proteins contain 4–6 AR domains stacked onto each other [190], whereas DARPins contain 2-3 internal ARs sandwiched between the N and C-terminal capping repeats. Each internal AR module consists of up to 27 defined framework residues and 6 potential protein-binding residues that form a β-turn, followed by two antiparallel helices and a loop connecting to the β-turn of the next repeat [190]. DARPins thus retain a relatively large binding interface, able to bind a wide range of targets down to the picomolar range [94].

DARPins exhibit some highly desirable traits, including a small size (15–18 kDa), high thermostability (T_m_ between 66–90 °C) [190], and high expression levels in *E. coli* (up to 200 mg/L shake flask culture, or 15 g/L with fermentation) [86,94]. DARPins also present many highly desirable traits specifically relevant to therapy, such as high tissue penetration, adjustable pharmacokinetics depending on their modification (e.g., PEGylation), high stability and solubility, as well as ease of production. Particularly their stability could allow them to be applied not just intravenously, but also topically, orally, through nasal administration, or inhalation. [191]. Furthermore, DARPins can be expressed multimerically at high yield, enabling bi-(or higher) specifics to be made, thus making targeted delivery or other therapeutic modalities possible [86]. Similar to other discussed scaffolds, small size decreases the half-life of DARPins, which can, however, be extended through PEGylation or conjugation to HSA [94,192]. Nonetheless, DARPins are promising candidates for broad therapeutic application, since they combine many desirable features into one molecule family [94,191].

To this date, at least four separate DARPin molecules have undergone clinical trials, with one (Abicipar pegol) already in Phase III [25]. Through multiple studies, DARPins have proved themselves strong candidates when it comes to targeted delivery. Therapeutics employing targeted delivery using DARPins have demonstrated an ability to reduce tumor growth, without causing fatal hepatoxicity, supporting their safety of use [193]. Finally, outside of clinical trials, DARPins have been demonstrated to be able to bind and neutralize the effect of soluble proteins, indicating that they may be promising modalities for toxin neutralization [194,195,196].

### 5.12. Fynomers

In 1989, Cooke and Perlmutter reported that amino acid residues 83–156 of the ~7 kDa Src-homology 3 domain of FYN tyrosine kinases contain two anti-parallel β-sheets, connected by two flexible, ligand-binding loops [197]. These loops can be engineered to obtain, or screened to discover, desired, ligand-binding specificity. This highly thermostable scaffold (T_m_ of 70 °C) was named a fynomer (Figure 1(12)) [198,199,200]. As fynomers are conserved across different species, including humans, mice, rats, and gibbons [93,201], they are non-immunogenic and can thus be utilized for therapeutic purposes. By fusing them with the Fc region, one can increase circulation half-life [93,202]. Other fusions might also lead to increased half-life, but are yet to be tested. Furthermore, they are easily recombinantly expressed in bacteria [198].

Unfused fynomers of ~7 kDa are expected to have good tissue penetration ability, as observed with nanobodies [104], adnectins [91], affitins [149], DARPins [191], and avimers [177], but even in its fused form, the molecular size is relatively small (depending on the fusion partner). This confers short circulation half-time, which may need to be addressed by increasing the molecular size by conjugation to e.g., HSA or targeting the fynomer to a serum protein by fusion to a targeting domain. This domain could be another fynomer connected by a linker or an Fc region with a linker to increase the molecular size. Furthermore, previous studies by Silacci et al. [199] showed that the longest linker tested between an IL-17A-targeting fynomer and an Fc domain showed the most efficient inhibition of IL-17A-mediated inflammation in vivo with an IC_50_ value of 21 pM.

Fynomers have been engineered as a bispecific format by fusion to antibodies, termed a “Fynomab”, enabling a reduced molecular size in comparison to a bispecific IgG, seeing as the fynomer can be fused directly to different antibody termini [203]. Fynomabs were demonstrated by targeting human epidermal growth factor receptor 2 (HER2)-overexpressing cancerous cells with a fynomer, while simultaneously recruiting T-cells via fusion to an anti-CD3 antibody [203]. The fynomer, targeting HER2, was selected specifically to only bind high-density HER2, and thereby not HER2 in low density on normal cells, demonstrating the strong engineerability of the approach. Later, in 2016, Silacci et al. developed a FynomAb (COVA332) which was able to simultaneously inhibit TNF & IL-17A [204]. This construct entered clinical trials (clinical trial ID: NCT02243787), but was later discontinued due to its safety profile.

## 6. Outlook

Plasma-derived antivenoms have historically been an effective treatment option for otherwise intractable envenomings. Today, however, there is an opportunity for innovation in the field via the application of technologies and approaches already well established in other fields. The development of recombinant, monoclonal, protein-based binders against pre-defined toxin targets through phage display selection is already underway and could be adapted to address the challenges seen for plasma-derived antivenoms (e.g., undefined polyclonality and low content of therapeutically active antibodies). Alternative scaffolds that exhibit significantly different properties to the more commonly used IgG scaffold are examples of promising therapeutic modalities, worthy of investigation in relation to next-generation envenoming therapies. There are now many different alternative protein-based binders, based upon bacterial or plant proteins (e.g., affibodies), non-antibody human proteins (e.g., DARPins) and antibody proteins themselves (e.g., nanobodies). These alternative binding proteins all exhibit different biochemical and pharmacokinetic profiles, which may be useful for future antitoxin development efforts. Some properties, such as high thermostability and low costs of production, are likely to be highly beneficial characteristics for future envenoming therapies that are to be distributed in impoverished regions of the developing world, while others (e.g., size and half-life) may need engineering before an optimal therapy can be derived. Furthermore, it may be the case that a combination of different scaffolds, such as oligoclonal mixtures of individual scaffolds from different classes or fusion proteins based on two or more different scaffold classes, will be of use in neutralizing whole venoms, as different toxins have different toxicokinetics, possibly requiring differential neutralization strategies. Consequently, it might become necessary to evaluate how different scaffolds interact with each other in solution, as this may affect their overall stability and efficacy. Alongside the current development of monoclonal antibody-based recombinant antivenoms, the inclusion of alternative binding protein scaffolds in the future of envenoming therapy deserves further investigation.
